# A CPW-Fed Circular Wide-Slot UWB Antenna with Wide Tunable and Flexible Reconfigurable Dual Notch Bands

**DOI:** 10.1155/2013/402914

**Published:** 2013-10-03

**Authors:** Yingsong Li, Wenxing Li, Qiubo Ye

**Affiliations:** ^1^College of Information and Communications Engineering, Harbin Engineering University, Harbin, Heilongjiang 150001, China; ^2^Communications Research Centre, 3701 Carling Avenue, Ottawa, Canada K2H 8S2

## Abstract

A coplanar waveguide (CPW)-fed circular slot antenna with wide tunable dual band-notched function and frequency reconfigurable characteristic is designed, and its performance is verified experimentally for ultra-wideband (UWB) communication applications. The dual band-notched function is achieved by using a T-shaped stepped impedance resonator (T-SIR) inserted inside the circular ring radiation patch and by etching a parallel stub loaded resonator (PSLR) in the CPW transmission line, while the wide tunable bands can be implemented by adjusting the dimensions of the T-SIR and the PSLR. The notch band reconfigurable characteristic is realized by integrating three switches into the T-SIR and the PSLR. The numerical and experimental results show that the proposed antenna has a wide bandwidth ranging from 2.7 GHz to 12 GHz with voltage standing wave ratio (VSWR) less than 2, except for the two notch bands operating at 3.8–5.9 GHz and 7.7–9.2 GHz, respectively. In addition, the proposed antenna has been optimized to a compact size and can provide omnidirectional radiation patterns, which are suitable for UWB communication applications.

## 1. Introduction

Ultra-wideband technology has been widely studied since the bandwidth of 3.1–10.6 GHz was released by the Federal Communications Commission (FCC) in 2002 for commercial applications due to its high data rate and low cost [1]. Afterwards, a number of techniques have been researched and developed both in industry and academia, including UWB antennas and UWB band-pass filters. A UWB antenna is one of the key components to realize a UWB system, and many UWB antennas with compact size have been investigated in recent years [2–18]. Within the UWB frequency band, several narrow-band systems sharing the same spectrum have been used for a long time, such as IEEE 802.11a WLAN system operating at 5.15–5.825 GHz, super high frequency (SHF) and satellite services operating at 4.5–5 GHz, IEEE 802.16 WiMAX system operating at 3.3–3.7 GHz, and ITU 8 GHz band operating at 7.725–8.275 GHz. In some cases, these narrow-band signals may interference with UWB systems or vice versa. To suppress these unwanted potential interferences, the traditional method is to add narrow-band band-stop filters at the end of the antenna or in the devices, which increases the complexity and cost of the devices [5, 6]. To design a UWB antenna with a filtering characteristic, cutting slots may be one of the simple, effective, and inexpensive methods. Antennas with small size and band rejection characteristics are also desirable and attractive. To implement the UWB antennas with filtering characteristic, a number of methods have been proposed by etching various slots on either radiation patches or ground planes, such as rectangle slot [5], C-shaped slot [6–8], pi-shaped slot [9], E-shaped slot [10], H-shaped slot [11], and U-shape slot [12]. Besides, combining the C-shaped slot and the U-shaped resonators [13] is also presented to provide better filtering function. The disadvantage of these etched slots is that they may leak electromagnetic waves, which will deteriorate the radiation patterns. Thus, some band-notched UWB antennas are realized by using stubs [14] and parasitic elements [15–17]. However, these antennas are only used either as multiband antennas or as band-notched UWB antennas, separately.

For these reasons, reconfigurability becomes another attractive method for designing a versatile UWB antenna combining characteristics of multiband and notch bands [18–23], which can be used as dual-notch band UWB antenna, single notch band UWB antenna, UWB antenna, or simply multiband antenna. Thus, a number of reconfigurable antennas have been proposed to meet one of these applications mentioned above [18, 20–23]. However, most of these antennas have complex structures, which is difficult to fabricate and adjust. One effective method is proposed to confirm the effectiveness of the reconfigurability by using ideal switches in recent years [18, 19, 21–25]. 

In this paper, a circular slot antenna with wide tunable dual band-notched characteristic and reconfigurable function is proposed and investigated in detail. The dual band-notched characteristic is realized by inserting a T-SIR inside the circular ring radiation patch and etching a PSLR in the CPW transmission line, and the corresponding center frequencies of these notch bands can be controlled by adjusting the dimensions of the T-SIR and the PSLR. The reconfigurability is realized by integrating three switches into the T-SIR and the PSLR. It is observed from the simulated and measured results that the proposed reconfigurable dual band-notched UWB antenna could operate from 2.7 GHz to 12 GHz with VSWR less than 2, except for the two notch bands at 3.8–5.9 GHz and 7.7–9.2 GHz for rejecting the unwanted C-, WLAN-, and X-band signals. In addition, the proposed antenna can also be used as a single band-notched UWB antenna or as a UWB antenna or as a multiband antenna by controlling the states of the switches ON or OFF. In a word, the designed antenna has band-notched characteristics, reconfigurable function, wide tunable notch bands, and omnidirectional radiation patterns, which makes it a good candidate for UWB communication applications.

## 2. Antenna Design

**Figure 1:**
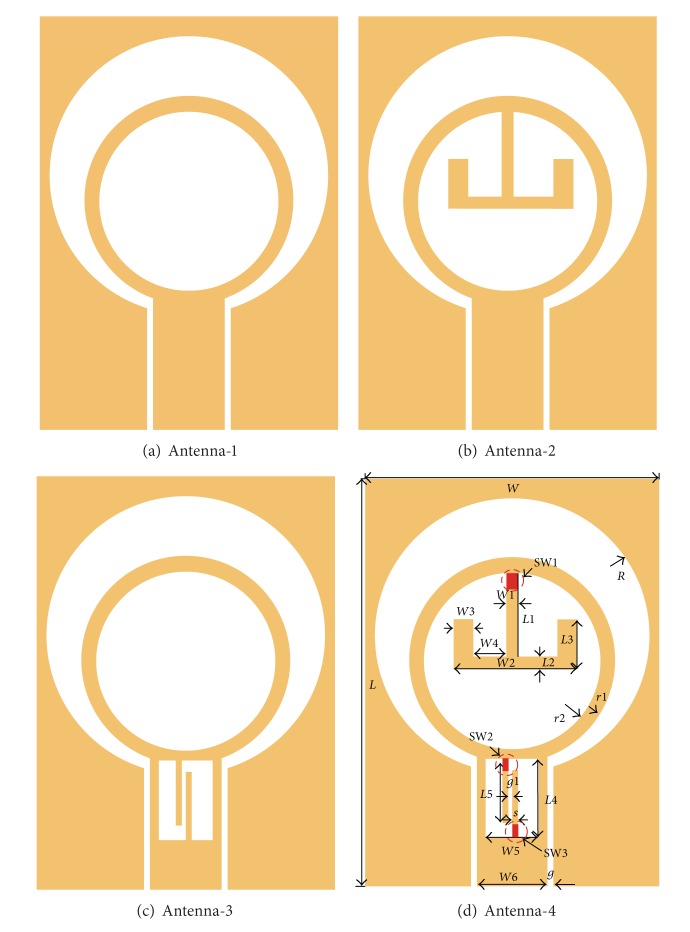
Design evolution and geometry of the proposed antenna.

Procedures of designing the proposed reconfigurable dual band-notched UWB antenna are shown in Figure 1. First, a UWB antenna without T-SIR and PLSR is illustrated in Figure 1(a) and is denoted as antenna-1. This antenna is comprised of a circular ring radiating element, a circular slot etched in the CPW ground plane, and a CPW ground plane together with the 50 Ohm CPW-fed structure. In the design, a substrate with a relative dielectric constant of 2.65 and thickness of 1.5 mm is employed. The 50 Ohm CPW feed structure consists of a transmission line with a width of *W*6 = 3.6 mm, and the gap between the transmission line and the ground plane is *g* = 0.2 mm. To implement the band-notched characteristic, two UWB antennas with single notch band are designed by using the T-SIR or the PSLR, shown in Figures 1(b) and 1(c), respectively. In Figure 1(b), the T-SIR is inserted inside the circular ring of antenna-1 to form one of the single band-notched antennas which is noted as antenna-2.  Figure 1(c) shows the other single band-notched UWB antenna with the PSLR etched in the CPW transmission line, which is defined as antenna-3. 

To obtain the dual band-notched characteristic, both the T-SIR and the PSLR are employed to realize the desired two stop bands at the same time, and the resulting dual band-notched UWB antenna is demonstrated in Figure 1(d) and is denoted as antenna-4. Additionally, three switches, namely, switches-1 (SW1), switches-2 (SW2), and switches-3 (SW3), are shown in Figure 1(d) for realizing the reconfigurability of the proposed antenna. In this design, the two notch bands can be designed independently. The notch band operating at 5.5 GHz WLAN band is implemented by using the T-SIR while the 8 GHz notch band is realized by using the PSLR, and the center frequency and the bandwidth of the two notch bands can be tuned by adjusting the dimensions of the T-SIR and the PSLR. In this study, the three ideal switches are used to evaluate the performance of the proposed reconfigurable notch band UWB antenna. To implement these switches, the presence of a metal bridge represents the ON state, while its absence represents the OFF state [18, 19, 21–25]. A commercial electromagnetic solver, High Frequency Structure Simulator (HFSS), is employed to model the antenna. The optimized parameters of the designed antenna are as follows: *L* = 32 mm, *W* = 24 mm, *L*1 = 4.5 mm, *W*1 = 0.6 mm, *L*2 = 0.6 mm, *W*2 = 4.8 mm, *L*3 = 2 mm, *W*3 = 1.0 mm, *L*4 = 4.5 mm, *W*4 = 1.1 mm, *L*5 = 4 mm, *W*5 = 2.6 mm, *W*6 = 3.6 mm, *g* = 0.2 mm, *s* = 0.3 mm, *g*1 = 0.3 mm, *R* = 11.6 mm, *r*1 = 6.6 mm, and *r*2 = 5.1 mm.

## 3. Performance of the Proposed Antenna

**Figure 2:**
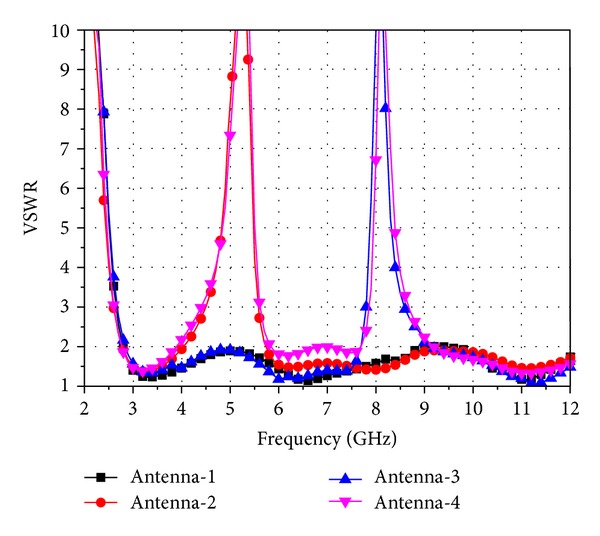
Band-notched characteristic of the four antennas while antenna-4 is with all the switches ON.

In this section, the performance of the proposed antenna, including the band-notched characteristic, effects of the key parameters, the reconfigurable characteristic, and the current density distributions, is studied by using HFSS.  Figure 2 shows the band-notch characteristic of the four antennas while antenna-4 is with all the switches ON. It can be seen that the antenna without the T-SIR and the PSLR, which is antenna-1, is a UWB antenna with a bandwidth of 9.3 GHz. It covers the entire UWB band. Antenna-2 with only the T-SIR is a UWB antenna with single notch band operating at 3.8–5.9 GHz, which is designed to prevent the potential interferences from C-band (4.4–5 GHz) for super high frequency (SHF) and satellite services and the interferences from IEEE 802.11a WLAN system (5.15–5.825 GHz). Antenna-3 with only the PSLR is a UWB antenna with a notch band operating at 7.7–9.2 GHz, which is used for mitigating the potential interferences from satellite communication systems (7.9–8.4 GHz) and ITU 8 GHz (7.725–8.275 GHz) applications. Antenna-4 with both the T-SIR and the PSLR, when all the switches are ON, is a dual band-notched UWB antenna with the two notch bands at 4–5.9 GHz (lower notch band) and 7.9–8.4 GHz (higher notch band), respectively. In this case, antenna-4 can reduce the potential interferences from the narrow band systems mentioned above, simultaneously. Thus, we can conclude that the lower notch band is achieved by using the T-SIR, while the higher notch band is realized by using the PSLR, and the two notch bands can be designed independently.

**Figure 3:**
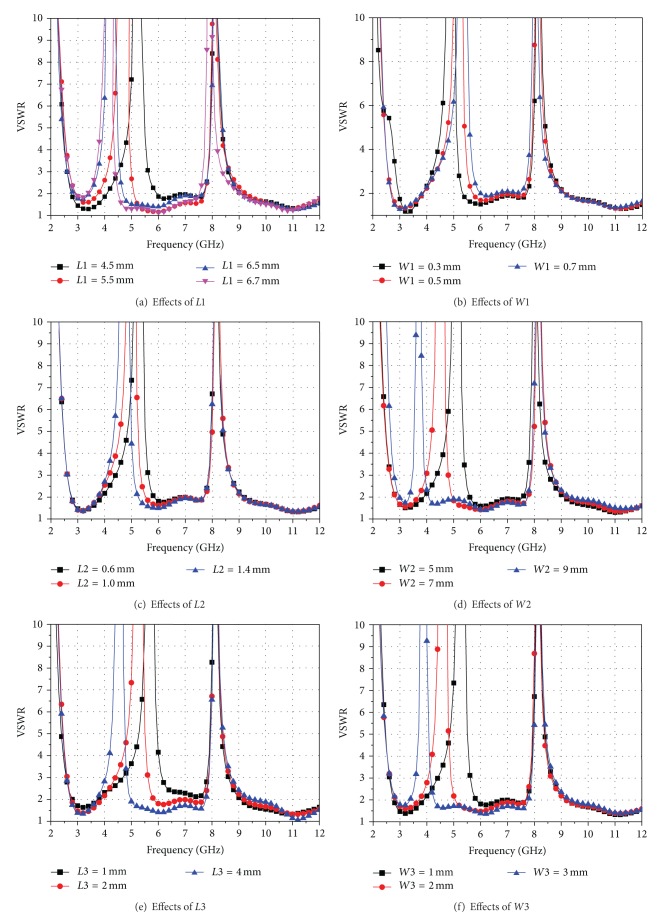

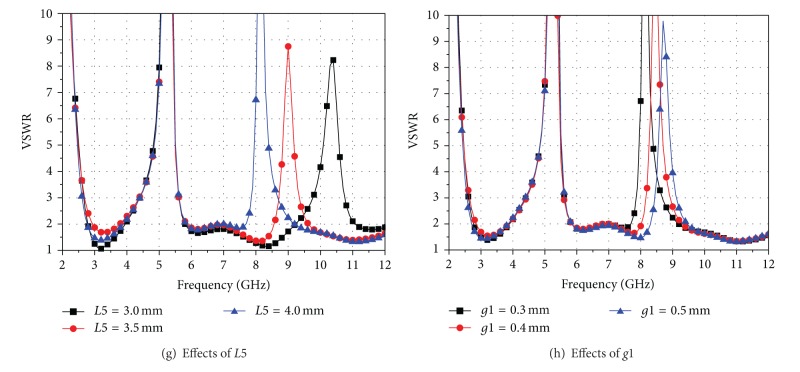
Parametric effects of antenna-4 with all switches ON.

Center frequencies of the dual notch bands can be tuned by adjusting the dimensions of the T-SIR and the PSLR. Thus, we study the effects of key parameters of the T-SIR and the PSLR to understand the tunable notch band characteristic of the antenna-4 with all the switches ON. Simulated results are obtained by using HFSS and they are displayed in Figure 3.  Figure 3(a) shows the effects of *L*1 on the antenna performance. We can see that the center frequency of the lower notch band moves to the low frequency with the increment of *L*1, while the higher notch band changes slightly. This is because the increased *L*1 lengthens the resonance length of the T-SIR, which also increases the current path along the T-SIR. It is worth noting that the lower notch band can be tuned widely from 3.3 GHz to 5.9 GHz, which may prevent the potential interference from IEEE 802.11a WLAN system operating at 5.15–5.825 GHz, super high frequency (SHF) and satellite services operating at 4.5–5 GHz, and IEEE 802.16 WiMAX system operating at 3.3–3.7 GHz. Effects of varying *W*1 on the antenna performance are shown in Figure 3(b), which reveals that the center frequency of the lower notch band moves to low frequency slightly with the increase of *W*1. However, the bandwidth of the notch band is reduced. Thus, *W*1 can be used not only to control the center frequency of the lower band, but also to adjust the bandwidth of the lower notch band.  Figure 3(c) describes effects of *L*2. With the increment of *L*2, the center frequency of the lower notch band shifts towards lower frequency and its bandwidth is also narrowed. Effects of *W*2 are demonstrated in Figure 3(d). It is found that the lower notch band moves to low frequency and its bandwidth is getting narrow with the increment of *W*2. This is because the increased *W*2 not only increases the resonance length of the T-SIR but also alters the coupling between the T-SIR and the radiating circular ring. Effects with varying *L*3 can be found in Figure 3(e), where it is observed that the center frequency of lower notch band moves to the low frequency with the increment of *L*3. Additionally, the impedance bandwidth between 5.9 GHz and 7.7 GHz is improved because of the strong coupling between the T-SIR and the circular ring.  Figure 3(f) reveals the effects of *W*3. It can be seen that the lower notch band shifts to low frequency with the increment of *W*3, while the higher notch band is almost constant. Thus, the lower notch band can be tuned by adjusting the parameters *L*1, *L*2, *L*3, *W*1, *W*2, and *W3* for desired results. Effects of *L*5 are described in Figure 3(g). It is observed that the higher notch band moves to the low frequency with the increment of *L*5. This is because the increased *L*5 increases the coupling between the parallel stubs, which changes the distributive capacitance and distributive inductance of the PSLR. In addition, the bandwidth of the higher notch band is narrowed. Effects of *g*1 are given in Figure 3(h), from where it can be seen that the higher notch band shifts to high frequency when we increase *g*1. This is because increasing *g*1 reduces the coupling of the parallel stubs. Thus, the higher notch band can be tuned by adjusting *L*5 and *g*1. Besides, the two notch bands can be designed independently by choosing the proper dimensions of the T-SIR and the PSLR.

**Figure 4:**
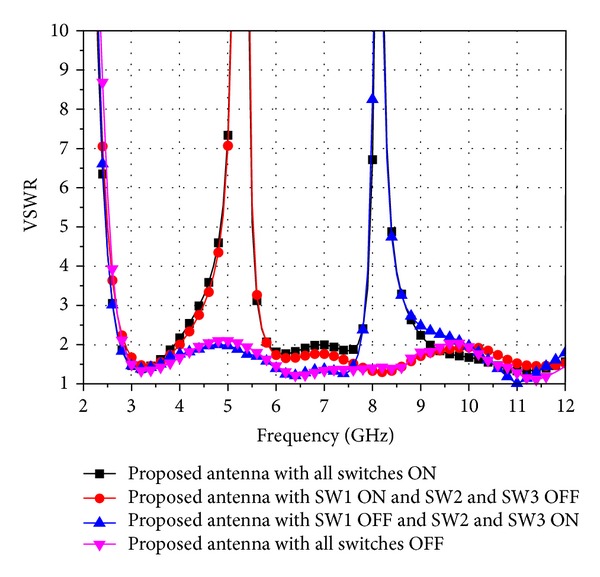
Reconfigurable characteristic of antenna-4.

In order to render the proposed antenna more useful, three switches are used to realize the reconfigurable function. In this investigation, SW2 and SW3 are turned ON or OFF simultaneously to keep the symmetry of the antenna so as to obtain good radiation patterns. Three ideal switches are employed to evaluate the performance of the proposed reconfigurable notch band UWB antenna. To approximate the switches, SW1 is replaced by a strip line with length of 0.5 mm and width of 0.6 mm, while SW2 and SW3 are represented by two strip lines with size of 0.5 mm × 0.3 mm. The performance of the reconfigurable characteristic is shown in Figure 4. As mentioned before, antenna-4 with all the switches ON is a dual band-notched UWB antenna. When SW1 is ON and SW2 and SW3 are OFF, the proposed antenna is a UWB antenna having a notch band operating at 3.8–5.9 GHz for filtering the unwanted signals from C- and WLAN band designated above. For the case of SW1 OFF and SW2 and SW3 ON, the antenna is a band-notched UWB antenna for reducing the potential interference from 7.7–10 GHz. When all the switches are OFF, the proposed antenna is a UWB antenna covering the 2.8–12 GHz frequency band. On the other hand, it has a little mismatch around 5 GHz because of the effects of the T-SIR and the PSLR. However, it can still be used for UWB communication applications due to its wide bandwidth and its good performance discussed above and later.

**Figure 5:**
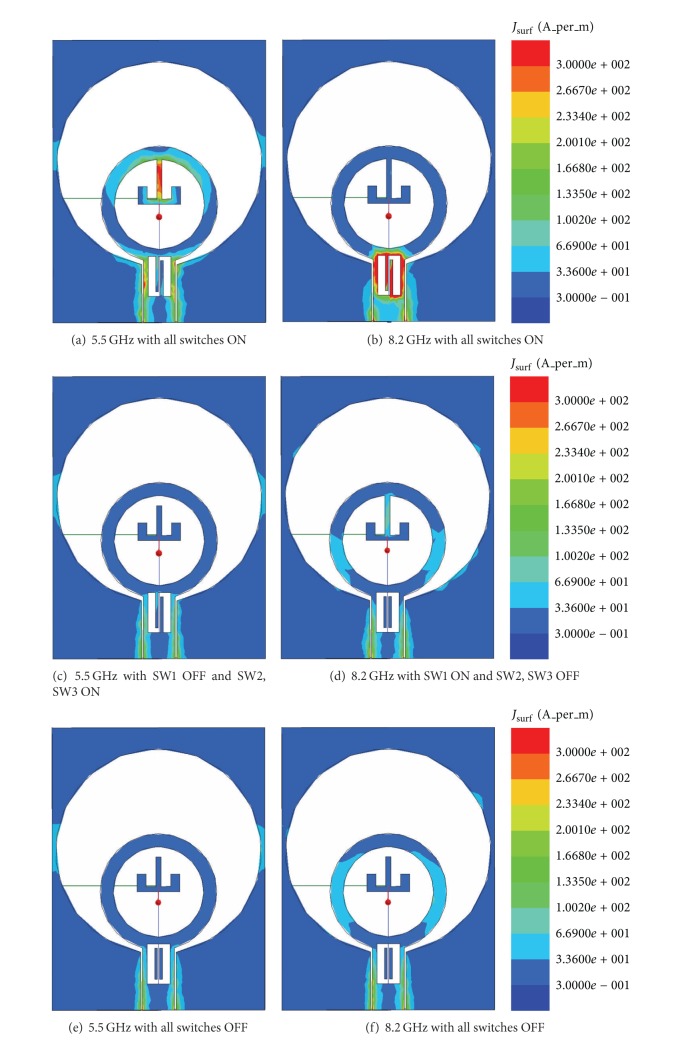
Current density distribution of the proposed reconfigurable dual notch band antenna.

To further study the principle of the proposed reconfigurable UWB antenna, current distributions of the antenna are investigated in the notch bands and are shown in Figure 5. Figures 5(a) and 5(b) illustrate the current distributions at 5.5 GHz and 8.2 GHz within the two notch band for antenna-4 with all the switches ON. In this case, the current at 5.5 GHz of the lower notch band concentrates on the T-SIR and the CPW-feed structure, while it is small on the radiation patch and the edge of the circular slot. At 8.2 GHz of the higher notch band, the current concentrates on the PSLR and the CPW-feed structure. Thus, the T-SIR and the PSLR act as good resonators at the corresponding rejection frequencies, which makes impedance mismatching achieve the desired notched bands.  Figure 5(c) reveals the current distribution at 5.5 GHz with SW1 OFF and SW2 and SW3 ON. The strong current has disappeared compared to Figure 5(a), which leads to turning the lower notch band OFF. In this case, at the higher notch band, the current on the PSLR is similar to that of Figure 5(b), which is not given in this paper.  Figure 5(d) shows the weak current of antenna-4 at 8.2 GHz of the higher notch band when the SW1 is ON and SW2 and SW3 are OFF. For 5.5 GHz at the lower notch band, the current on the T-SIR is similar to that of Figure 5(a) and is not given herein. Such current distributions are caused by SW2 and SW3 switching off the PLSR. Therefore, the higher notch band has disappeared. Figures 5(e) and 5(f) demonstrate the current distributions at 5.5 GHz and 8.2 GHz, respectively, when all switches are OFF. It is found that the strong currents are disappeared and they mainly flow along the CPW-feed structure and the edge of the circular wide slot. Thus, we can conclude that the switches can control the modes of the proposed reconfigurable antenna, making it either a dual band-notched UWB antenna or a single band-notched UWB antenna or a conventional UWB antenna. 

## 4. Results and Discussions

**Figure 6:**
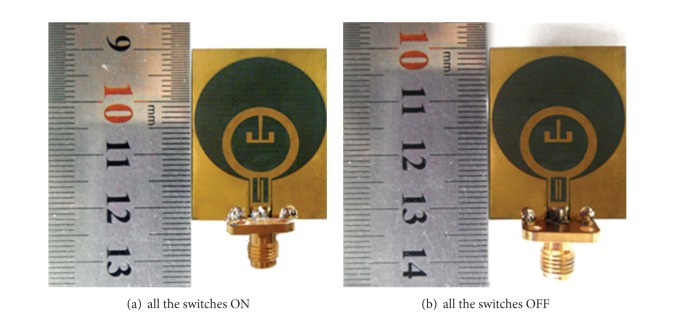
Prototypes of the fabricated antennas.

**Figure 7:**
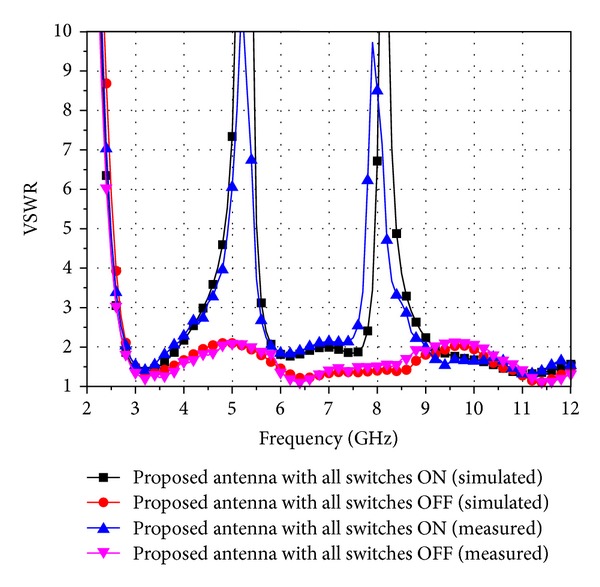
Comparison of the simulated and measured VSWR of the fabricated antennas.

To validate the designs of the proposed reconfigurable dual band-notch UWB antenna, antenna-4 with all the switches ON or OFF has been fabricated and tested. To be consistent with the simulation setting of HFSS, the presence of a metal bridge in the fabricated antenna corresponds to the ON state while its absence represents the OFF state. The fabricated antennas are shown in Figure 6, and the comparison of the measured VSWRs with the simulation results is presented in Figure 7, which helps to verify the accuracy of the HFSS simulation. It clearly shows that the proposed antenna-4 does perform as a dual band-notch UWB antenna when all the switches are ON. The two notch bands are as expected at 5.5 GHz and 8 GHz. With all the switches OFF, the proposed antenna functions as a UWB antenna, whose impedance bandwidth is 9.3 GHz, ranging from 2.7 GHz to 12 GHz or even higher. Thus, it can cover the entire UWB band. Hence, the reconfigurable dual band-notch UWB antenna can be realized by controlling the states of the appropriate switches. 

**Figure 8:**
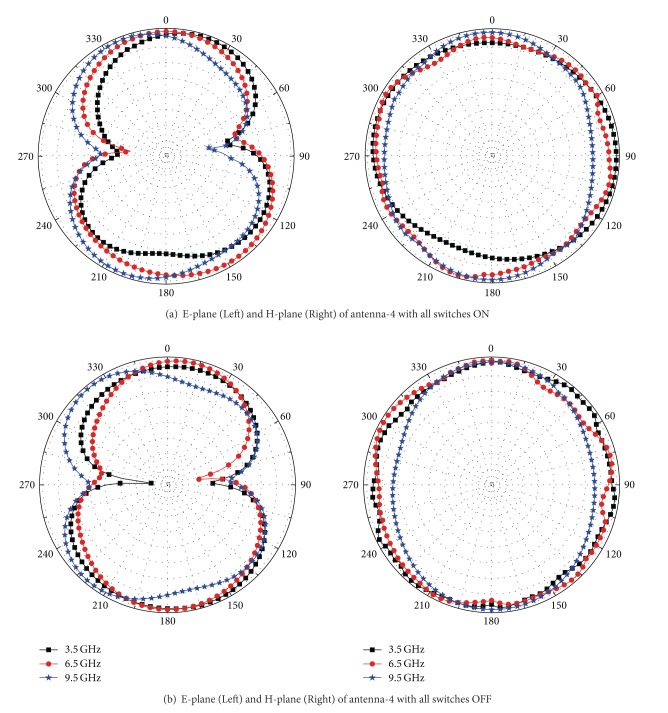
Radiation patterns of the fabricated antennas.

**Figure 9:**
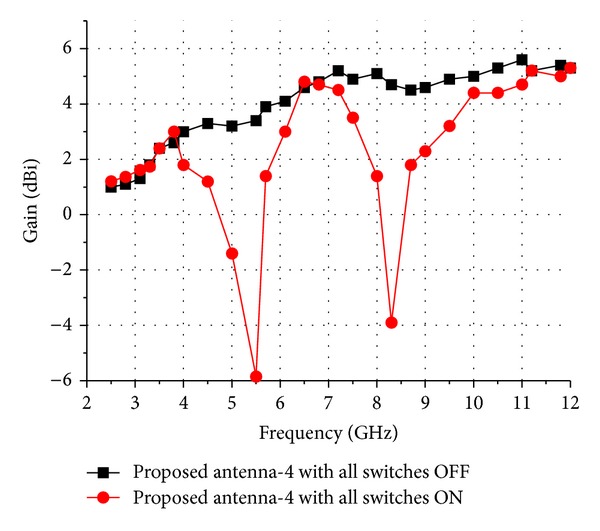
Gain of the fabricated antennas.

The measured results shown in Figure 7 agree well with the simulated ones, although there are minor differences. These discrepancies between the measured and simulated results may be attributed to tolerance errors in fabrication and manual welding inaccuracies. Radiation patterns of the fabricated reconfigurable dual band-notch UWB antenna with all switches ON or OFF at 3.5 GHz, 6.5 GHz, and 9.5 GHz have been measured and shown in Figures 8(a) and 8(b). It can be seen that the antenna has omnidirectional radiation characteristics in the H-plane and monopole-type radiation characteristics in the E-plane. The radiation patterns deteriorate slightly at high frequencies, and this can be attributed to the radiation of T-SIR inserted inside the radiation ring and the PSLR embedded in the CPW-fed transmission line. The gains of the fabricated antennas are derived by comparing them to a horn antenna in the chamber, and the measured results are shown in Figure 9. As discussed above, the energy at the two frequency rejection bands is not radiated so that the peak gains drop at these notched bands. We can see that the gains of the antennas with all the switches either ON or OFF are stable over the UWB operating band, with the exception of the 5.5 GHz WLAN- and C-notch bands and the 8 GHz notch band when all the switches are ON. The gain decreases by approximately −5.8 dBi in the WLAN band and by −3.9 dBi in the X-band. 

## 5. Conclusion

In this paper, a reconfigurable dual band-notch UWB antenna has been designed, and its performance has been verified experimentally. The two notch bands have been realized by using the T-SIR and the PSLR. The results show that the antenna can be used either as a dual band-notched UWB antenna with two notch bands at 5.5 GHz and 8 GHz or as a single band-notched UWB antenna with the notch located either at 5.5 GHz WLAN band or at 8 GHz X-band. Additionally, it can be used as a conventional UWB antenna, if desired. The reconfigurable characteristics can be controlled by turning appropriate switches on and off. The proposed antenna, which has a wide impedance bandwidth, dual notch band functions, and reconfigurable characteristics, is suitable for UWB applications, where interference suppression at one or two specified frequencies is desired.
